# Some Notes on the Philippines

**Published:** 1900-01

**Authors:** Frank S. Bourns

**Affiliations:** Atlanta, Ga.; Late Major and Chief Surgeon U. S. V., Late President of Board of Health, Manila, P. I.


### SOME NOTES ON THE PHILIPPINES.

BY FRANK S. BOURNS, M.D.,

Late Major and Chief Surgeon U.S.V., Late President of Board of Health, Manila, P. I.

Compared with other tropical regions the Philippine Archipelago possesses a healthful climate. W·th the exception of Luzon and Mindanao, none of the islands possess an area of over 5,000 or 6,000 square miles, and even in these two a considerable area is subject to the direct influence of the prevailing winds from the ocean.

Being a mountainous (and volcanic) region, every island possesses highlands, and in but few places are considerable areas of low or swampy lands found, nowhere such swamps as girt the great island of Borneo.

Extending as they do over 1,000 miles of latitude, some variations are seen between the northern and southern islands, but everywhere a distinctly tropical climate prevails. In the highlands, especially of central and north Luzon, a delightful temperate climate is found, the extreme being 32° and 75° F. The two prevailing seasons are of course the wet and the dry. During the former the rains usually prevail two or three hours every afternoon, although sometimes it will rain steadily for a week. At such times life is not altogether agreeable. The dry season is not a markedly dry time, as rains occur every few days. Coming during the months corresponding to our winter, it is the pleasantest time of the year.

The diseases prevalent are those usually found in tropical countries. The islands are free, however, from some of the most dreaded ones. Yellow fever is unknown, cholera is normally not found, but is sometimes introduced from India, Singapore and Hongkong, when it usually becomes epidemic. The last epidemic was in 1889, but was not nearly so severe as that of 1882. Bubonic plague has so far been kept out by strict quarantine regulations, and it is to be hoped that this dread disease may never find its way into the islands. That it would flourish, especially in Manila and the larger cities, there is no doubt. Of the prevailing diseases I shall speak briefly, not in detail, as we should soon have the report of Dr. Flexner and Dr. Barker of Johns Hopkins. These two gentlemen spent several months in Manila in the military and city hospitals, studying the diseases found there, and their report will be of great interest and value to the profession. Of first importance are the intestinal diseases and malaria. Of the former we find acute and chronic dysentery, amebic dysentery and milder forms, as diarrhea, both acute and chronic. These prevail throughout the year, but are most common during the hot season, beginning with May and extending through June, July and August. With the exception of the amebic form, these diseases are largely due to indiscretions in the manner of living. By proper attention to diet and drink and by observation of ordinary hygienic rules, these diseases can, in my opinion, be avoided in most instances. During a residence of three years and a half, most of the time traveling about the provinces, often in the midst of the jungle, my companion, Professor Worcester, and myself suffered but once or twice with slight attacks of diarrhea. Overeating, eating of indigestible or poorly cooked foods and excessive indulgence in alcoholic drinks, and, of course, the use of impure or unboiled water, are the causes of the great majority of cases. Lack of attention to slight intestinal troubles, as simple diarrheas, contributes to more severe sickness. During four and a half years residence in this region, it was always a wonder to me that Europeans and Americans kept well at all. They would give excellent advice to the newcomer about the use of the abdominal bandage, and then invite him to a late dinner where rich foods and strong liquors would be served in abundance. We never used the abdominal bandage, but we did eat simple, nourishing food and avoided alcoholic drinks, and I believe that we had as little intestinal trouble as we would have had in an equal period of time in this country.

Malaria in all its forms is found in the different islands. Some regions are notoriously malarial, as the island of Mindoro. For nearly a year my companion and myself were free from the disease, but a visit to this island brought us both down. On a later visit, with six native servants, we visited the interior near Mt. Halcon. Within a week each and every member of our party of eight had been attacked.

The usual quotidian and tertian forms are most common, but some regions, usually small in extent, are noted for the prevalence of the pernicious form. In the autumn months, in certain regions usually comparatively free from malaria, the estivo-autumnal form prevails. The disease is of course most prevalent in jungle regions, and especially where new ground is being broken. Many of the older regions are almost or quite free from the disease. Such a place was by chance selected for the first landing and encampment of the American troops, and during the month that this place (Camp Dewey, near Paranaque) was occupied almost no cases of malaria occurred among the 7,000 or 8,000 troops.

Many regions at one time noted for their unhealthfulness have been freed from malaria by the adoption of modern methods of sanitation. Such a place is Sulu (Jolo), where sanitation and the introduction of a good supply of water changed a notoriously bad place into one so healthful that it was selected as the site of a Spanish military hospital. Smallpox is an endemic disease throughout the archipelago, and in the months of March, April, May and June usually increases to the point where in this country it would be called epidemic.

The people seem to have no fear of it and take no precautions whatever to prevent its spread. I have often seen children with the eruption out playing on the village streets with their companions. On the death of a person the funeral feast is often had as usual, and the uncovered body is carried through the streets to the church and from there to the cemetery. Isolation and disinfection are unknown.

Under the Spanish régime, vaccination was compulsory in theory, but in practice was far from it.

That the disease can be eradicated by proper methods of vaccination and isolation there is no doubt. In Manila, under most unfavorable conditions, with the population massed in the central districts of the city, we succeeded in stamping out a threatened epidemic in a few weeks' time. Compulsory vaccination, disinfection and isolation accomplished this, and these same methods applied on a larger scale would accomplish the same result in the country at large. That obstacles would have to be overcome there is no doubt, as the natives are somewhat superstitious, believing that vaccination at a time when smallpox is prevalent is sure to bring the disease on the person vaccinated. They are likewise unaccustomed to the isolation of the sick, and oppose this measure; but a little tact and firmness soon overcome their objections, and when once convinced of the utility of the measures adopted, they cooperate with commendable willingness. This at least was the experience in Manila, and I see no reason why it should not be the same in the provinces. Excellent vaccine can be cheaply procured from the water buffalo and apt persons easily found to carry on the actual vaccination. The vaccination of eight or ten million people scattered over a large area is of course a large task, but it presents no insurmountable obstacles, and under American control I expect to see it accomplished in the near future.

Another medical problem of interest is that of leprosy. This disease is found in many of the islands, being most common in the central or Visayan district. There are but two hospitals, one at Manila and one at Cebu. In the former there have been during the past year from 100 to 125 patients. Before the American occupation there were nearer 200, but during the siege the hospital, situated on the outskirts of the city, was abandoned by the friars in charge, the inmates being left to shift for themselves.

As soon as the city came under American control every effort was made to return these liberated lepers to the hospital, and as fast as they were discovered this was done. That the whole 200 were not found was due to the fact that many of them had returned to their native towns outside the city, beyond our control.

At Cebu there is a hospital capable of accommodating about 100 patients. Around this hospital temporary quarters for 100 more have been erected. But this is not nearly sufficient, as on a visit to that city in June I was informed that there were probably 500 lepers in the region, 300 of them unprovided for. They could be seen in groups of five or six, begging on the streets. This was under the control of the native government, not the American, as at that time our authorities had not taken over the control of civil affairs.

The whole problem is one that demands immediate attention from our authorities. The solution of this question would be in my opinion the adoption of the plan in vogue in the Hawaiian Islands. Of the large number of small islands in the group, it would be easy to select a suitable one, centrally located, where a leper colony could be established. Here they could be allowed to raise their crops, carry on fishing and such other occupations as they desired. In this way contributing materially to their own support. A hospital with proper faciilties would provide for those in the more abandoned stages of the disease.

Of other diseases may be mentioned beriberi (at present epidemic in the islands), tuberculosis, rheumatism and typhoid fever. The latter three are found in about the proportion found in this country. The former is an endemie disease, which fortunately for us, is confined to the native population. Better sanitary conditions, better wages, and therefore better food and intelligent control will probably render this of minor importance.

Venereal diseases are common in the larger towns and cities, especially the seaports. In Manila, a peculiarly virulent form of chancroid is much more commonly seen than is the true syphilitic chancre. It is almost always followed by buboes in one or both sets of inguinal glands, many of them going on to suppuration. Skin diseases are common, particularly the so-called dhobee itch. This disease, which can be easily controlled if taken early by the use of a 1 to 3 oz. formalin solution, caused a great deal of annoyance among our troops. Officers and enlisted men alike were attacked, as the infection usually comes through the clothes. All washing is done in the streams near the city or possibly at the home of the washerwoman, with water brought from these streams. The clothes are never boiled, the prospect of infection being the best. As a guess I would say that 50 per cent. of the army had this troublesome skin disease. True itch is not uncommon. Among the Moros of the southern islands skin diseases, such as eczema and especially ichthyosis, are quite commonly found.

In conclusion I would state that I see no reason why Americans should not live in the Philippines in comfort and in the enjoyment of good health. When the condition of the country becomes more settled, so that sanitation can be improved, Manila should be a very pleasant city. Sooner or later, under American control, some of the temperate highlands, such as the Benguet region, will, with improved means of communication, be brought within easy reach of the city; and then there will be established summer resorts where the families of Americans and Europeans may pass the months of extreme heat, June, July and August. When these changes are accomplished life in the Philippines will be not only safe but pleasant as well.

